# Editorial: Pathophysiological Interrelationship Between Obesity, Metabolic Diseases, and Cancer

**DOI:** 10.3389/fonc.2021.755735

**Published:** 2021-09-14

**Authors:** Manuel D. Gahete, Riccarda Granata, Raúl M. Luque

**Affiliations:** ^1^Maimónides Institute of Biomedical Research of Córdoba (IMIBIC), Córdoba, Spain; ^2^Department of Cell Biology, Physiology and Immunology, University of Córdoba, Córdoba, Spain; ^3^Reina Sofía University Hospital, Córdoba, Spain; ^4^CIBER Pathophysiology of Obesity and Nutrition (CIBERobn), Córdoba, Spain; ^5^Division of Endocrinology, Diabetes and Metabolism, Department of Medical Sciences, University of Turin, Turin, Italy

**Keywords:** obesity, cancer, metabolic syndrome, tumor, metabolism

Obesity and metabolic syndrome are chronic endocrine-metabolic diseases that represent emerging global epidemics and capital health problems for the global population (1–3). Unfortunately, the presence of obesity and metabolic syndrome increases the risk of developing more severe endocrine pathologies [e.g. insulin resistance or type-2 diabetes (T2D)], cardiovascular complications, and some cancer types (4–8). Indeed, emerging evidence indicates that cancer development, progression, and aggressiveness are strongly influenced by the presence of metabolic alterations such as obesity and metabolic syndrome, wherein 20% of cancer incidence seems to be attributable to obesity (9). However, the precise association of the different indicators of metabolic dysregulation (body weight, obesity, metabolic syndrome, hyperglycemia, etc.), as well as the molecular, cellular, and endocrine-metabolic mechanisms underlying the pathophysiological association between metabolic abnormalities and cancer, remain to be fully elucidated.

This Research Topic compiles eight original research, clinical trial, review, and systematic review articles that advance our knowledge of and shed light on the complex pathophysiological association between obesity, metabolic diseases, and cancer. Firstly, the review by Scully et al. provides a detailed summary of the epidemiological evidence linking the different components of obesity and metabolic syndrome and their associated comorbidities (BMI, insulin resistance, hyperinsulinemia, dyslipidemia, and T2D) and different cancer types, including colon, breast, prostate, and liver. These epidemiological data are in line with those reviewed by the International Agency for Research on Cancer (IARC) and the World Cancer Research Fund/American Institute for Cancer Research (WCRF/AICR) that indicated an association of excessive body fat with increased risk of certain cancer types (10, 11). The review by Scully et al. recapitulates evidence from preclinical and cross-sectional clinical studies, suggesting a substantial interaction between adipose tissue and cancer, which could contribute to the obesity-associated promotion of tumor growth. This relationship is especially evident in some cancer types such as breast cancer and esophageal adenocarcinoma, which are particularly affected by the obesogenic status. Examples of this close association are presented in the mini review of Bhardwaj and Brown, which explores the role of adipose tissue in obesity as a driver of breast cancer growth and development, and the review by Elliot and Reynolds, that recapitulates the epidemiological evidence supporting the association between visceral obesity and metabolic syndrome with esophageal adenocarcinoma. Moreover, the original research article by Ardesch et al. demonstrates the importance of dissecting out the different components of the metabolic dysregulations and the diverse indicators of obesity (BMI, adiposity, etc.) to appropriately analyze their association with cancer incidence as they demonstrate that, in the case of lung cancer, there is a non-linear association between BMI and the risk of lung cancer while waist circumference, waist-to-hip ratio, and other body shape indexes are positively and linearly associated with the risk of this cancer type.

Therefore, the risk of cancer development in patients with obesity and/or other metabolic alterations should be specially monitored by more proactive screening programs or by implementing prevention/reversal strategies aimed to reduce the cases of different cancer types associated with metabolic dysregulations. As demonstrated by the clinical trial article by Sung et al., subjects who were willing to undergo colorectal cancer screening tended to accept a subsequent breast and prostate cancer screening, indicating the effectiveness and compliance of a one-stop service for cancer screening among asymptomatic subjects, which could be specially relevant in patients with metabolic complications. In this line, the reviews by Bhardwaj and Brown and Elliot and Reynolds also recapitulate current evidence indicating the impact of obesity-related strategies, such as pharmacological interventions, physical activity, and weight lost in cancer incidence and/or development. However, these results should be interpreted considering the metabolic status of the patients, in that the systematic review by Lin et al., indicated that, in patients with T2D, weight change achieved by hypoglycemic agents or strategies over short and medium periods are not associated with incidence of most neoplasms, although it was effective in decreasing the incidence of prostate, bladder, and uterine neoplasms.

Finally, the review articles by Scully et al., Bhardwaj and Brown, and Elliot and Reynolds also examine the mechanisms underlying the pathophysiological relationship between obesity, metabolic diseases, and cancer. Among the proposed drivers of this association, they reviewed the implication of key hormonal systems (insulin/IGF-I, estrogens, or sex steroids), as well as certain adipokines, circulating lipids, and other adipose tissues-derived mediators, such as immune cells (macrophages) and cytokines/interleukins. The accumulated evidence assigning a key role to novel mechanisms related to tumor and adipose tissues microenvironment or to the putative role of the microbiome in this pathophysiological interrelationship is also emphasized. Altogether, it seems evident the existence of common drivers that could be mediating the pathological crosstalk between obesity/adiposity/metabolic syndrome and different cancer types, as clearly illustrated by the review of Ku and Cheng, focused on the impact of the master regulator Activating Transcription Factor 3 (ATF3) in metabolism and cancer. ATF3 is a stress-induced transcription factor that exerts pivotal roles in modulating immune homeostasis and glucose and adipose tissue regulation, as well as shared actions on cell proliferation and metastasis in breast, prostate, colon, lung, and liver cancers. In the last study of this Research Topic, Frugé et al. integrates most of the aspect mentioned above, including the relationship between obesity and prostate cancer, weight lost interventions, microbiota, lipid profile, and changes in classic and novel molecular markers to demonstrate a multifarious association that should be further explored in future studies to finely unveil this pathological crosstalk.

Altogether, the articles included in this Research Topic add novel evidence and further support to the multifaceted pathophysiological interrelationship between obesity, metabolic diseases, and cancer. However, these articles also suggest that this is not a universal and linear relationship and should instead be analyzed by taking into account multiple parameters and considering the type of cancer, the different metabolic complications of the patients, and other confounding factors (age, race, sex, etc.). Similarly, it is also evident that the molecular, cellular, and endocrine-metabolic mechanisms that trigger the pathophysiological association between metabolic dysregulations and cancer are not fully elucidated and need further investigation.

## Author Contributions

MG, RG, and RL contributed to conception of the Research Topic and the Editorial. MG wrote the first draft of the Editorial. RC and RL revised and completed the Editorial. All authors contributed to the article and approved the submitted versión.

## Conflict of Interest

The authors declare that the research was conducted in the absence of any commercial or financial relationships that could be construed as a potential conflict of interest.

## Publisher’s Note

All claims expressed in this article are solely those of the authors and do not necessarily represent those of their affiliated organizations, or those of the publisher, the editors and the reviewers. Any product that may be evaluated in this article, or claim that may be made by its manufacturer, is not guaranteed or endorsed by the publisher.
